# Dioscin Inhibits Virulence Factors of* Candida albicans*

**DOI:** 10.1155/2018/4651726

**Published:** 2018-11-26

**Authors:** Longfei Yang, Xin Liu, Lili Zhong, Yujie Sui, Guihua Quan, Ying Huang, Fang Wang, Tonghui Ma

**Affiliations:** ^1^Jilin Provincial Key Laboratory on Molecular and Chemical Genetics, The Second Hospital of Jilin University, Changchun 130041, China; ^2^Eye Center, The Second Hospital of Jilin University, Changchun 130024, China; ^3^College of Oceanology and Food Science, Quanzhou Normal University, Quanzhou 362000, China; ^4^College of Basic Medical Sciences, Dalian Medical University, Dalian 116044, China; ^5^Institute of Traditional Chinese Medicine, Nanjing University of Chinese Medicine, Nanjing 210023, China

## Abstract

*Candida albicans* infections present a heavy burden upon public health, with only a few drugs available, while biofilms formed by* C. albicans* worsen this situation. Dioscin has antitumor, anti-inflammatory, and hepatoprotective effects, and this study was conducted to evaluate the effects of dioscin on the biofilm formation and development, as well as other virulence factors of* C. albicans* such as morphological transition, adhesion, and extracellular secreted phospholipase. Our results showed dioscin inhibits these virulence factors and has low cytotoxicity against mammalian cells. Considering protective effects of dioscin against damage on liver and kidney, dioscin may be used as a potential candidate for antifungal development.

## 1. Introduction

*Candida albicans* severely influences health of immunocompromised individuals, as this opportunistic fungal pathogen could overgrow rather than grow under immune surveillance in these hosts. This kind of uncontrollable existence causes infections in oral cavity and gastrointestinal and urogenital tract, as well as the life-threatening bloodstream infections [1]. Patients with invasive* Candida* infections are often associated with high mortality (about forty percent), despite antifungal therapy [2, 3]. The paucity of available drugs and the unsatisfying profiles of such drugs, such as toxicity, resistance, and low bioavailability, recapitulate the necessity to develop new antifungals [4].

As a virulence factor, biofilms formed by* C. albicans* are a complicated three-dimensional structure, with yeast, hyphal, and pseudohyphal cells embedded in extracellular matrix. Biofilms provide a shelter for cells within from attack by antifungals and host immune system [3]. Therefore,* C. albicans* infections associated with biofilms are often refractory and recurrent. Examples are biofilms on surfaces of medical devices such as catheters and joint prosthesis [3, 5]. The removal of devices often results in therapeutic failure and the fee is high [5]. This highlights again the need for development new antifungals, especially those active against biofilms.

Dioscin is a steroidal saponin that could be isolated from medicinal herbs and vegetables of* Dioscorea* genus (family Dioscoreaceae), such as* Dioscorea zingiberensis*,* Dioscorea nipponica*,* Dioscorea panthaica*,* Dioscorea spongiosa*, and* Dioscorea cayenensis* [6–10]. Dioscin has been reported to have pharmacological activities against various kinds of tumors such as gastric, colon, and gallbladder cancer and osteosarcoma [11–14]. Meanwhile, this saponin has been documented with antiviral activities against adenovirus, vesicular stomatitis virus, and hepatitis B virus [15]. Dioscin also exerts anti-inflammatory activities through TLR4/MyD88/NF-*κ*B signaling [16]. The antihyperuricemic activity of dioscin was reported to be through enhanced excretion and decreased reabsorption of uric acid in rat model of hyperuricemia [9]. Protective effects of dioscin against liver, lung, and renal damage, as well as pulmonary fibrosis and cardiotoxicity (induced by doxorubicin), have also been reported recently [17–21]. Dioscin could also promote the regeneration of liver, which was mediated by the activation of Notch1/Jagged 1 signaling [22]. Although the antifungal activity of dioscin against* C. albicans*, as well as other* Candida* species and* Saprolegnia parasitica*, has been reported [7, 10, 23], the effects of dioscin on the virulence factors of* C. albicans* have yet not been tested. In the present study, we evaluated the antifungal effects of dioscin on the biofilm formation and development, as well as virulence factors such as adhesion, morphological transition, and extracellular enzyme production.

## 2. Materials and Methods

### 2.1. Chemicals and Strains

*C. albicans* SC5314,* C. albicans* ATCC10231,* Candida glabrata* ATCC2001, and* Candida parapsilosis* ATCC22019 were obtained from China General Microbiological Culture Collection Center (CGMCC). All these strains were maintained on YPD (yeast extract 1%, peptone 2%, dextrose 2%) medium and stored at -80°C. Before each test, a colony from fungal strains subcultured twice on YPD agar was transferred into YPD medium and incubated overnight at 30°C with a speed of 140 rpm for propagation.

Dioscin was bought from Solarbio Life Sciences. N-acetyl-D-glucosamine (GlcNAc), XTT, MTT, menadione, propidium iodide (PI), and RPMI-1640 medium were bought from Sigma.

### 2.2. Antifungal Susceptibility Tests

Broth microdilution methods were employed to test the antifungal activities of dioscin against fungal strains according to CLSI-M27-A3 guidelines [24]. These assays were done in triplicate and repeated three times.

### 2.3. Time-Killing Assay

*C. albicans* SC5314 cells collected from overnight cultures in YPD medium were resuspended in 1640 medium to reach a concentration of 10^6^ cells/mL. Cell suspensions were challenged with different concentrations of dioscin at 30°C. At indicated time points, an aliquot from each treatment was taken and plated on YPD agars after serial dilution. After incubation for 24 h, numbers of colony forming units (CFUs) on YPD agars were counted. This assay was conducted in triplicate and repeated three times.

### 2.4. Yeast-to-Hypha Transition

1640 medium, Spider medium (nutrient broth 1%, mannitol 1%, K_2_HPO_4_, pH7.2), and GlcNAc medium (GlcNAc 0.4%, HK_2_PO_4_ 0.3%, peptone 0.5%) were used to explore the effects of dioscin on the yeast-to-hyphal morphological transition.* C. albicans* SC5314 cell suspension at a concentration of 1 x 10^6^ cells/mL in each medium was transferred into wells of 96-well plates and dioscin in DMSO was added to each well to obtain different final concentrations (0, 1, 2, and 4 *μ*g/mL). After incubation at 37°C for 4 hours, the morphologies of cells exposed to different concentrations of dioscin were recorded by microscope.

### 2.5. Biofilm Test

Biofilms of* C. albicans* SC5314 were formed in 96-well plates according to the well-established methods [25]. To test the inhibitory effect of dioscin on the biofilm formation, different final concentrations of dioscin were coincubated with* C. albicans* cells at 37°C for 24 h, which was followed by XTT reduction assay. Meanwhile, in another 96-well plate under the same conditions, cells in each well were determined by CFU counts as mentioned above.

As for the inhibitory effect on preformed biofilms, 24-hour old biofilms in 96-well plates were washed with PBS and exposed to fresh 1640 medium containing different concentrations of dioscin for another 24 h, which was followed by XTT reduction assay. Wells with only medium were set as blank while wells containing biofilm without drug exposure were set as negative controls. These assays were performed in triplicate and repeated three times.

### 2.6. XTT Reduction Assay

To evaluate the viability of cells in biofilms, 100 *μ*L of sterile XTT solution (0.5 mg/mL in PBS) supplemented with 1*μ*M menadione was added into each well, preceded by washing with PBS to remove free-floating fungal cells. After incubation at 37°C for 2 h in dark, 70 *μ*L of the supernatant of each well was transferred into a new 96-well plate and the optical density at 490 nm (OD_490_) of each well was detected by a microplate reader (VarioSkan, thermo, Germany). The viability of biofilm in wells = (OD_490  treated_-OD_490  blank_)/(OD_490  negative_-OD_490  blank_) × 100%.

### 2.7. Confocal Laser Scanning Microscope (CLSM) Analysis

To study the influence of dioscin on 3D structures of* C. albicans* biofilms, biofilms formed in the presence of dioscin were stained with Calcofluor White (CFW, 50 *μ*g/mL) and subjected to CLSM analysis (Olympus FV1000, Japan).* Z*-axis scanning was employed while the number of photo-slices (step size: 2 *μ*m) varied among different biofilms. 3D graphs of biofilms were reconstructed by Imaris 7.2.3 software (Bitplane, Switzerland).

### 2.8. Adhesion Test

The capacity of* C. albicans* SC5314 cells to adhere to polystyrene surfaces was determined by calculating viability of the residual cells in 96-well plates after exposure to dioscin and washing with PBS [26]. After cells were treated with dioscin for 1.5 h at 37°C, cells-contained wells were washed with PBS and XTT solution was added to each well to determine the viability of cells challenged with dioscin.

To investigate whether the effects of dioscin on adhesion were due to the decrease in cell numbers caused by dioscin, viable cells in each well were counted. In brief, after treatment with different concentrations of dioscin for 1.5 h as aforementioned, all cells in each well were resuspended with sterile PBS, collected by vigorous aspiration using a pipette, and serially diluted and plated on YPD agars. After incubation at 37°C for 24 h, CFUs on each agar plate were counted. These experiments were performed in triplicate and repeated three times.

### 2.9. Phospholipase Assay

Egg yolk emulsion agar was used to assess the effect of dioscin on phospholipase production of* C. albicans* [27]. Briefly, 1 *μ*L of fungal cell suspension was added to the center of egg yolk emulsion agar supplemented with different concentrations of dioscin and plates were incubated at 37°C for 96 h to allow the precipitation zones and colonies to form on agars. The Pz value was used to quantify the enzymatic production. Pz = *d*_colony_/*d*_conlony+precipitation_ where* d* means the diameter of colony or precipitation.

### 2.10. PI Influx Assay

After incubation at 37°C for 4 h,* C. albicans* cells in 1640 medium (10^6^ cells/mL) were stained with PI (10 *μ*M) and subjected to confocal microscope analysis.

### 2.11. Cytotoxicity Assessment

MTT assay was employed to evaluate the toxicity of dioscin against mammalian JEG3 cells [28]. Confluent cells grown at 37°C in a humidity of 5% CO_2_ were challenged with different concentrations of dioscin for 24 h, followed by MTT reduction assay.

### 2.12. Statistical Analysis

For each assay, at least three independent tests were performed to generate results expressed as mean + standard deviations. GraphPad Prism 6.02 was used to produce figures and to calculate the significances of differences between groups (treatment vs control) through Student's* t*-test.

## 3. Results

### 3.1. Antifungal Susceptibility

From the results of antifungal susceptibility tests (Table 1), dioscin showed significant antifungal activities against* Candida* species (*C. albicans*,* C. glabrata*, and* C. parapsilosis*), with MICs ranging from 2 to 4 *μ*g/mL and MFCs ranging from 4 to 8 *μ*g/mL. The activities of dioscin were fungicidal.

Because of the similar antifungal profiles of two* C. albicans* strains displayed, we chose the widely used standard strain SC5314 for further investigations.

### 3.2. Time-Kill Kinetics

**Figure 1:**
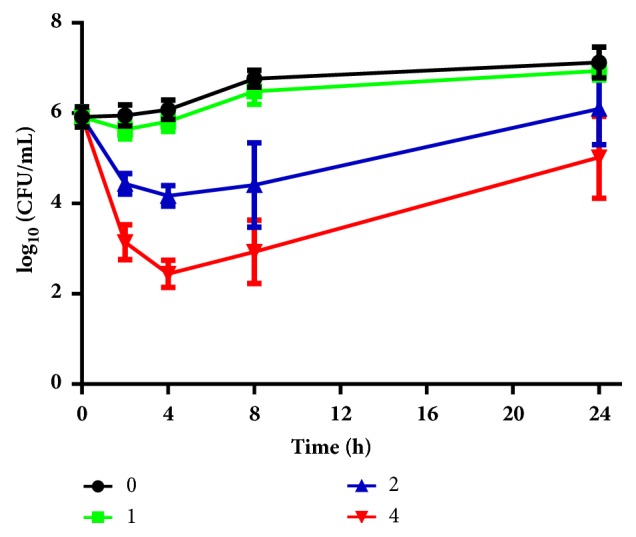
Time-kill kinetics of different concentrations of dioscin.* C. albicans* cell suspension with different concentrations of dioscin was grown at 30°C, 140 rpm. At indicated time points, 100 *μ*L solution was taken from cultures and the viable cells within were determined by plating on YPD agars. CFU counts were performed after incubation for 24 h at 37°C. Assays were performed in triplicate and repeated three times.

As a complementary assay to antifungal susceptibility tests, time-kill assays were performed (Figure 1), and it was found that, in the first four hours, treatment with 4 *μ*g/mL dioscin caused a reduction of 3 log_10_ CFU/mL compared to the initial inoculum, indicating a fungicidal effect of dioscin on* C. albicans*, which was consistent with antifungal susceptibility results. However, since the 4th hour, the CFU of samples treated with 4 *μ*g/mL dioscin started to recover, indicating a short-term fungicidal effect.

### 3.3. Dioscin Inhibits Biofilm Formation and Development

**Figure 2:**
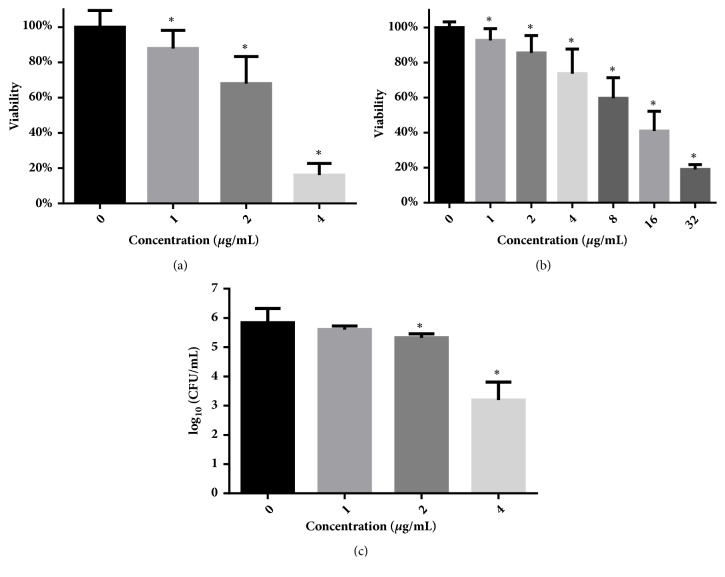
Antibiofilm activities of dioscin against biofilm formation (a) and development (b) of* C. albicans*. The duration of treatment for both formation and development of biofilms was 24 h. Viability of biofilms treated was detected by XTT reduction assay. (c) Under biofilm-forming conditions, dioscin treatment did reduce cell numbers at sub-MIC concentrations. CFUs were counted by diluting cells in each well and plating on YPD agars. *∗* means* p* < 0.05.

**Figure 3:**
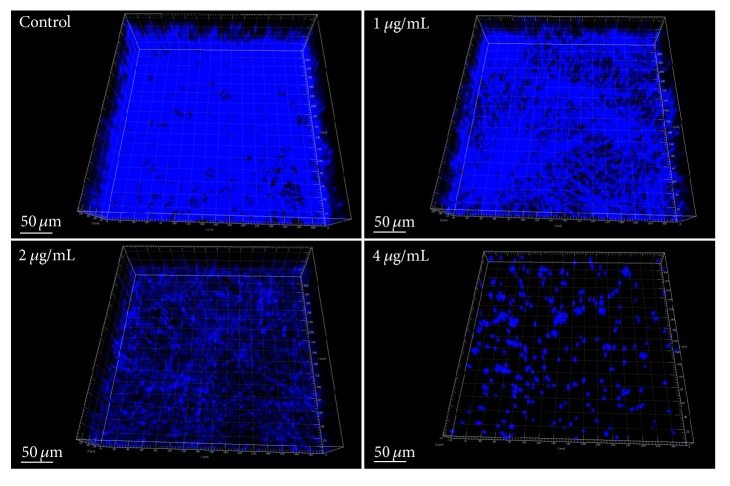
Reconstructed 3D graphs of biofilms formed under treatment with 0, 1, 2, and 4 *μ*g/mL of dioscin for 24 h. Cells in biofilms were stained with Calcofluor White. Biofilms formed on polystyrene surfaces under dioscin exposure were recorded by confocal microscope and visualized by Imaris 7.2.3.

As shown in Figure 2(a), dioscin concentrations ranging from 1 to 4 *μ*g/mL suppressed 10% ~ 80% of biofilm formation, while this concentration band could only reduce the viability of cells in mature biofilms by less than 30% (Figure 2(b)). Only at a high concentration of 16 *μ*g/mL could this compound decrease more than 50% of cell viability of preformed biofilms (Figure 2(b)). The inhibitory effect of dioscin on biofilm formation could also be validated by confocal microscope, as biofilms that formed in the presence of increasing concentrations of dioscin showed decreased heights and proportion of filamentous cells (Figure 3). To quantify the cell numbers in biofilm after washing, CFU measurements were performed. As shown in Figure 2(c), dioscin decreased the cell numbers in biofilms formed in the presence of dioscin. This could also be confirmed by CLSM analysis in Figure 3, where, with increasing concentration of dioscin, the cell distribution in biofilms becomes sparser.

### 3.4. Dioscin Inhibits the Adhesion of* C. albicans*

**Figure 4:**
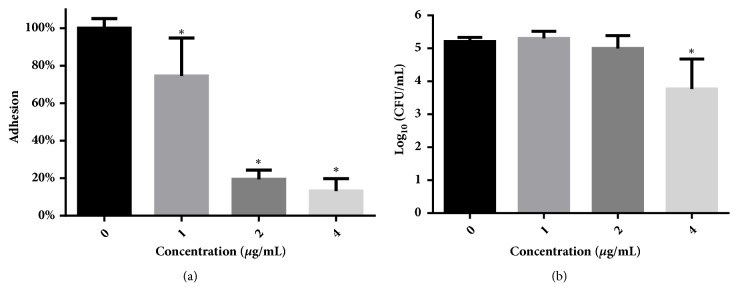
Dioscin inhibits the adhesion of* C. albicans* cells to polystyrene surfaces of 96-well plates. (a) Adhesion was calculated through comparing the residual viability of drug-treated cells left on surfaces after wash with those of drug-free controls, determined by XTT assay. (b) The inhibition of dioscin on adhesion was not completely due to the alterations in cell numbers. After 1.5 h incubation, cells in each well were collected and diluted for CFU counting. *∗*,* p* < 0.05.

Based on our determinations that dioscin inhibited the biofilm formation, we speculated that dioscin may influence the adhesion of* C. albicans* cells to polystyrene surfaces since adhesion is the first step to form biofilms. To test our conjecture, we employed XTT assay to quantify the adhesion using viability of residual cells having undergone treatment with dioscin and PBS washing. As shown in Figure 4(a), dioscin inhibited the adhesion of* C. albicans* cells to polystyrene surfaces in a dose-dependent manner. To test whether this inhibition was due to the changes in cell numbers caused by dioscin, all cells of each group (adherent and nonadherent) were measured by CFU counts. As shown in Figure 4(b), although 4 *μ*g/mL of dioscin reduced the viable cells significantly, 1 and 2 *μ*g/mL of dioscin did not, suggesting that the reduced adhesion on polystyrene surfaces was not completely due to the alternations in cell numbers. In other words, dioscin did demonstrate inhibition on the adhesion of* C. albicans* cells on polystyrene surfaces.

### 3.5. Dioscin Inhibits Yeast-to-Hyphal Transition of* C. albicans*

**Figure 5:**
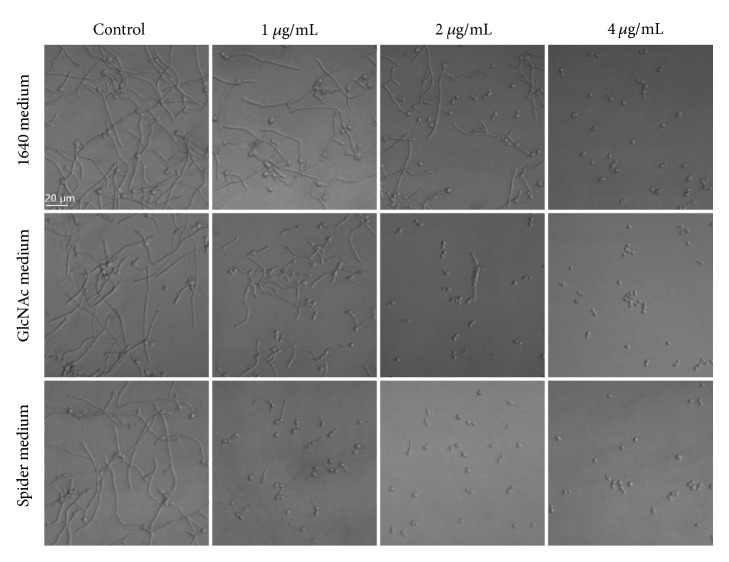
Dioscin inhibits morphological transition in different media.* C. albicans* cells (10^6^ cells/mL) in 1640 medium, GlcNAc medium, and Spider medium were exposed to different concentrations of dioscin for 4 h at 37°C before the morphological changes were captures by inverted microscope.

To test the inhibitory effects of dioscin on yeast-to-hyphal transition, we used three kinds of hyphal-inducing media, namely, 1640, Spider, and GlcNac medium. As shown in Figure 5, dioscin inhibited the morphological transition of* C. albicans* SC5314, although the extent of inhibition varied in different media.

### 3.6. Dioscin Inhibits the Production of Phospholipase

**Figure 6:**
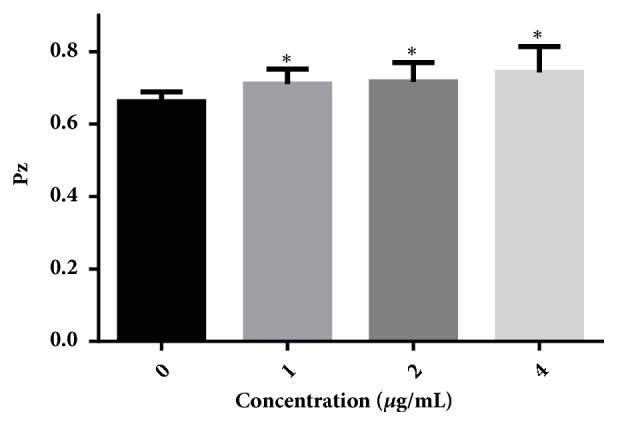
Dioscin inhibits the production of phospholipase secreted by* C. albicans* SC5314. In the phospholipase assay, a larger Pz value indicates less enzyme production. *∗*,* p* < 0.05.

To investigate whether dioscin has an inhibitory effect on the production of* C albicans* phospholipase, egg yolk emulsion agars were employed. In this assay, a smaller Pz value means a higher enzyme production. As shown in Figure 6, 1-4 *μ*g/mL of dioscin could decrease the production of phospholipase by* C. albicans* cells in a concentration-dependent manner.

### 3.7. Dioscin Causes Damage in Cell Membrane

**Figure 7:**
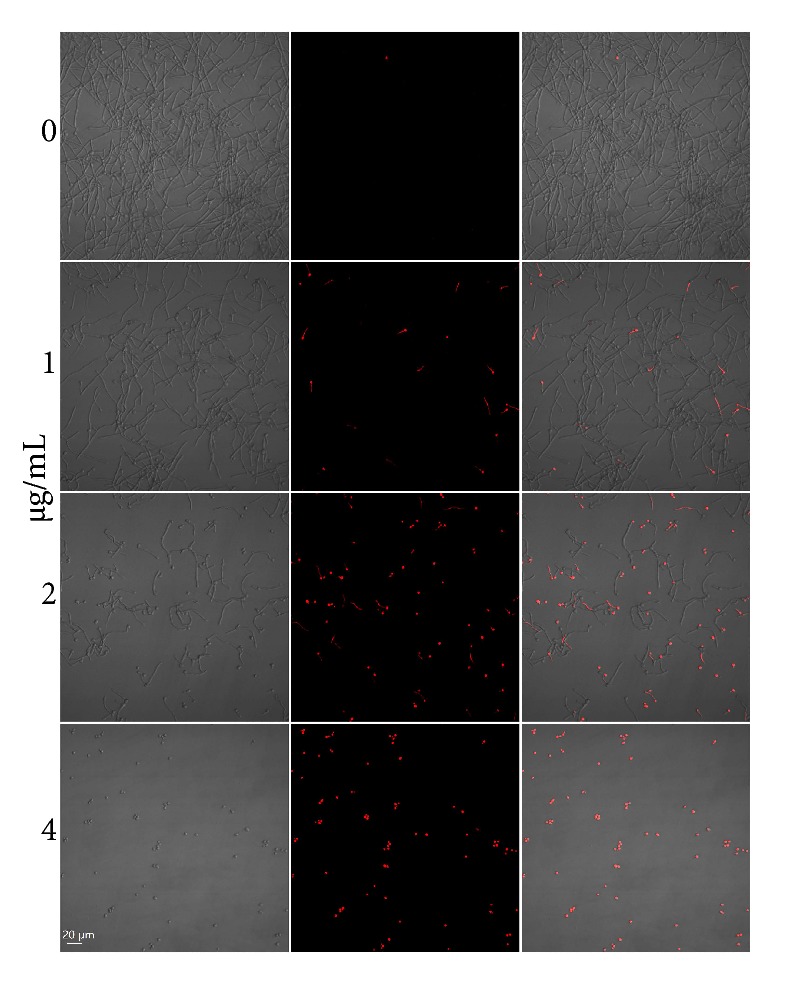
PI staining indicates that dioscin caused plasma membrane permeability. After being incubated with different concentrations of dioscin for 4 h,* C. albicans* cells were stained with PI and recorded by a confocal microscope.

Cell membrane integrity was evaluated by staining with PI, a membrane-impermeable fluorescent dye that could not gain access into cell unless the plasma membrane is disrupted. As shown in Figure 7, incubation with dioscin could induce compromised cell membrane integrity in a dose-dependent manner, indicted by increased cells with red fluorescence.

### 3.8. Cytotoxicity of Dioscin

**Figure 8:**
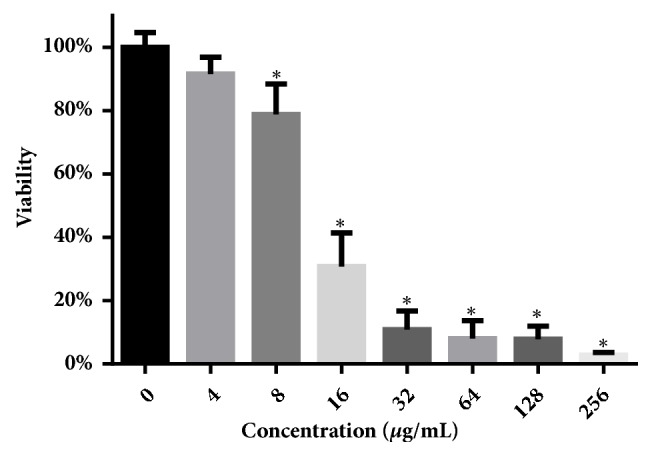
Cytotoxicity of dioscin against human JEG3 cells. After dioscin treatment for 24 h, MTT assay was performed to assess the inhibitory effect of dioscin on cell proliferation. *∗*,* p* < 0.05.

Cytotoxicity against human JEG3 cells was assessed using MTT assay. Although JEG3 cells treated with dioscin showed decreased viability with a half maximal inhibitory concentration (IC_50_) of about 13 *μ*g/mL (as shown in Figure 8), the IC_50_ was higher than MICs against* C. albicans* cells, implying relatively low cytotoxicity.

## 4. Discussion

The burden on public health imposed by* C. albicans* infections and the limited antifungal drugs available against fungal pathogens, as well as the toxicity and drug resistance associated with conventional drugs, necessitate the development of new antifungal agents [29]. Recent years have seen increased enthusiasm in combating diseases with natural products, and dioscin is one of them [1, 21, 30]. The various protective effects of dioscin have been reported by others [18, 21], and the aim of this study was to investigate the effects of dioscin on virulence factors of* C. albicans*.

From antifungal susceptibility tests, we got a different MIC (4 *μ*g/mL) against* C. parapsilosis* ATCC22019, compared with previously reported MIC (11.3±4.6 *μ*g/mL) by others, which probably resulted from the different media (1640 vs YPD medium) and initial inoculum concentrations (2x10^3^ vs 10^6^ cells/mL) used [7].

Since saponin extract of* Dioscorea panthaica* Prain* et* Burk rhizomes which contains dioscin has been demonstrated to have antifungal effects against* C. albicans*, our results are consistent with these previous reports [11, 31–33]. However, 4 *μ*g/mL of dioscin could only reduce the viable cells in the first four hours, followed by an increase in viable cells, which is different with our previous research on dioscin-containing extract [33]. At 16 *μ*g/mL (4MIC), dioscin also showed similar curves (data not shown). This might indicate that in the extract there may exist other components with antifungal activity that synergize with dioscin, exerting a more powerful and longer fungicidal effect. The short-term fungicidal activity of dioscin was also seen in* n*-dodecanol, an alcohol which could induce transient fungicidal effect in* Saccharomyces cerevisiae* and could synergize with* trans*-anethole (at sub-inhibitory concentration) to produce a long-lasting fungicidal effects [34].

Although dioscin has been reported to have antifungal activity against* C. albicans* [7, 10], our results demonstrated for the first time that dioscin has antibiofilm activity, with IC_50_ for biofilm formation and development less than 4 *μ*g/mL and 16 *μ*g/mL, respectively. Given that azoles, such as fluconazole and voriconazole, have their BMIC90s (concentrations that inhibit 90% biofilm formation) high above 256 and 1000 *μ*g/mL and that concentration required for AmB to inhibit biofilm formation is 8-31 times above its MIC [35], dioscin may make a promising candidate for antifungal drug development and optimization. Besides the viability analysis through XTT assay, results obtained by confocal microscope also confirmed the inhibitory effect of dioscin on biofilm formation of* C. albicans*. Combining results from XTT reduction, CFW staining, and CFU assays, the antibiofilm activity of dioscin may be due to the antifungal effects of dioscin against* C. albicans*. This is similar to the antifungal drugs caspofungin and lipid formulation of Amphotericin B, which showed efficacy on* C. albicans* biofilms [35].

Adhesion to abiotic or biotic surfaces is the first step to initiate the biofilm formation, thus making compounds inhibiting the adhesion attractive for antifungal development [36]. Multiple reported studies showed that compounds with antibiofilm activity also inhibit the adhesion process, such as filastatin, magnolol, honokiol, and dracorhodin perchlorate [28, 37, 38]. Dioscin has similar antiadhesion effects to those compounds.

The capacity of* C. albicans* to transit from yeast to hyphae phenotype has been considered as an important virulence factor during pathogenesis [39]; the influence of dioscin on the transition was investigated. In the three hyphal-inducing media, dioscin could significantly inhibit the hyphal growth. The different extent of hyphal inhibition may result from different inhibitory activity on distinct signaling pathways activated by individual stimulus [40], which need to be further validated.

As an important kind of extracellular hydrolase of* C. albicans*, phospholipase plays key roles in invasion and colonization by degrading components of epithelial cells. Mutants of phospholipase showed decreased colonization in major organs of infected mice, indicating attenuated virulence [39, 41]. In our research, dioscin reduced the production of phospholipase of* C. albicans*, consistent with other compounds with antifungal activity, such as dracorhodin perchlorate and quercetin [28, 42].

The surfactant activity of dioscin might underlie the antifungal effects through membrane-disruptive mechanism [7, 43]. In the current study, the membrane-damaging effects of dioscin have also been affirmed by increased PI influx induced by dioscin, as shown in Figure 7, which was in line with previous results published by others [7].

Considering the relatively low cytotoxicity of dioscin against human JEG3 cells, as well as the absence of obvious adverse effects* in vivo* via oral administration [14], dioscin may be effective against* C. albicans* infections* in vivo*, though much more need to be done.

Despite the fact that dioscin has good antifungal and antivirulence activities and relative low cytotoxicity, however, dioscin has a very low bioavailability through oral administration (approximately 0.2%) [43], which might limit the oral use of dioscin for systemic infections. Another drawback of dioscin is the poor solubility in aqueous solution, but there are already improvements designed to form copolymer micelles that facilitate the solubility [44]. Of note, the dioscin in mixed micelles demonstrated better antitumor activities against mammalian tumor cell lines, as compared to free dioscin, probably resulting from enhanced uptake by cells. The pharmacokinetic profiles have also been improved by this design, making it a potential drug-delivering system [44].

The brighter part of developing dioscin as a lead is that many analogous compounds have already been synthesized, making it more easier and more well-founded to decipher the structure-activity correlation and to optimize the chemical structures for antifungal therapy [45–47]. Another advantage of dioscin as an antifungal agent is that this compound has been used as a vital material for synthesizing other drugs such as cortisone [23], which means that dioscin could be easily used for industrial production. The third advantage of developing dioscin as an antifungal agent is safety or at least very low toxicity, considering that dioscin-containing herbs and vegetables have been consumed for centuries and that dioscin-containing DA-9801 has been approved by FDA for treating diabetic neuropathy [18, 48, 49].

## 5. Conclusion

In conclusion, dioscin showed significant antifungal activities against several* Candida* species tested through fungicidal effects. Dioscin could also inhibit the virulence factors of* C. albicans*, such as yeast-to-hyphal transition, extracellular phospholipase production, adhesion to abiotic surfaces, and biofilms formation. At higher concentrations, dioscin could even reduce the viability of preformed biofilms. Given the low toxicity dioscin has [18, 48, 49] and the readiness for structure-activity analysis and structural optimization [45–47], dioscin may make a promising candidate for antifungal therapies.

## Figures and Tables

**Table 1:** Antifungal profiles of dioscin against *Candida* species.

Fungal strains	MIC (*μ*g/mL)	MFC (*μ*g/mL)	MFC/MIC	Antifungal effects
*C. albicans* SC5314	4	4	1	Fungicidal
*C. albicans* ATCC10231	4	4	1	Fungicidal
*C. glabrata* ATCC2001	2	4	2	Fungicidal
*C. parapsilosis* ATCC22019	4	8	2	Fungicidal

## Data Availability

The data used to support the findings of this study are included within the article. The more detailed data are available from the first author upon request.
